# Isolation of ACE2-dependent and -independent sarbecoviruses from Chinese horseshoe bats

**DOI:** 10.1128/jvi.00395-23

**Published:** 2023-09-28

**Authors:** Hua Guo, Ang Li, Tian-Yi Dong, Hao-Rui Si, Ben Hu, Bei Li, Yan Zhu, Zheng-Li Shi, Michael Letko

**Affiliations:** 1 CAS Key Laboratory of Special Pathogens and Biosafety, Wuhan Institute of Virology, Chinese Academy of Sciences, Wuhan, China; 2 Savaid Medical School, University of Chinese Academy of Sciences, Beijing, China; 3 Paul G. Allen School for Global Health, Washington State University, Pullman, Washington, USA; Loyola University Chicago, Maywood, Illinois, USA

**Keywords:** coronavirus, sarbecovirus, zoonosis, cross-species transmission, bat

## Abstract

While the spike proteins from severe acute respiratory syndrome coronaviruses-1 and 2 (SARS-CoV and SARS-CoV-2) bind to host angiotensin-converting enzyme 2 (ACE2) to infect cells, the majority of bat sarbecoviruses cannot use ACE2 from any species. Despite their discovery almost 20 years ago, ACE2-independent sarbecoviruses have never been isolated from field samples, leading to the assumption these viruses pose little risk to humans. We have previously shown how spike proteins from a small group of ACE2-independent bat sarbecoviruses may possess the ability to infect human cells in the presence of exogenous trypsin. Here, we adapted our earlier findings into a virus isolation protocol and recovered two new ACE2-dependent viruses, RsYN2012 and RsYN2016A, as well as an ACE2-independent virus, RsHuB2019A. Although our stocks of RsHuB2019A rapidly acquired a tissue-culture adaption that rendered the spike protein resistant to trypsin, trypsin was still required for viral entry, suggesting limitations on the exogenous entry factors that support bat sarbecoviruses. Electron microscopy revealed that ACE2-independent sarbecoviruses have a prominent spike corona and share similar morphology to other coronaviruses. Our findings demonstrate a broader zoonotic threat posed by sarbecoviruses and shed light on the intricacies of coronavirus isolation and propagation *in vitro*.

**IMPORTANCE:**

Several coronaviruses have been transmitted from animals to people, and 20 years of virus discovery studies have uncovered thousands of new coronavirus sequences in nature. Most of the animal-derived sarbecoviruses have never been isolated in culture due to cell incompatibilities and a poor understanding of the in vitro requirements for their propagation. Here, we built on our growing body of work characterizing viral entry mechanisms of bat sarbecoviruses in human cells and have developed a virus isolation protocol that allows for the exploration of these understudied viruses. Our protocol is robust and practical, leading to successful isolation of more sarbecoviruses than previous approaches and from field samples that had been collected over a 10-year longitudinal study.

## INTRODUCTION

With the increase of coronaviruses crossing the species barrier into humans and causing severe diseases over the last 20 years, significant effort has been invested into understanding coronaviruses in diverse animals, globally. The first viral relatives of severe acute respiratory syndrome coronavirus (SARS-CoV) were discovered in *Rhinolophus* bats in 2005, demonstrating these animals are a natural reservoir for the sarbecovirus subgenus of the betacoronaviruses (1, 2). However, in comparison to SARS-CoV, these bat sarbecoviruses contained numerous polymorphisms in their spike glycoprotein—the viral protein responsible for binding cell receptor molecules and mediating viral invasion into host cells. Later, cell-culture-based studies with these bat sarbecoviruses showed that although their spike proteins were not compatible with some human receptors, exchanging their spike genes with the SARS-CoV spike allowed for the viruses to replicate in cell culture—demonstrating that cell entry is a primary species barrier for bat sarbecoviruses (3). The identification of bat sarbecoviruses that could bind angiotensin-converting enzyme 2 (ACE2), the same receptor as SARS-CoV, has led to the overall assumption that bat sarbecoviruses that do not use this receptor pose little threat of zoonosis to humans.

In a broad screen of sarbecovirus entry, we found several host cell entry phenotypes that are determined by the presence or absence of deletions within the receptor-binding domain (RBD) of the spike glycoprotein (4). Clade 1 RBDs do not contain any deletions and are capable of binding ACE2 from multiple species; clade 2 RBDs contain two deletions and do not use ACE2; and clade 3 and 4 RBDs contain a single deletion but are capable of binding ACE2 more specifically from their host species (4 – 12). The first bat sarbecoviruses discovered were clade 2 viruses and any attempts to isolate them from field samples have failed (1, 2). We recently showed that a high concentration of trypsin could facilitate *in vitro* entry and replication of pseudotyped virus particles and recombinant sarbecoviruses containing clade 2 RBD spike proteins (4, 13). Many other viruses have been shown to replicate in the presence of trypsin, including several gastrointestinal coronaviruses with uncharacterized host receptors (14 – 17). Taken together, these findings suggest that some clade 2 bat sarbecoviruses may also have the capacity to infect human cells, which is a prerequisite for cross-species transmission to humans.

Here, we further optimized our methods for propagating clade 2 sarbecoviruses in culture for viral isolation from field samples. We successfully isolated one clade 2 RBD sarbecovirus as well as two new clade 1 RBD sarbecoviruses from *Rhinolophus sinicus* fecal samples collected between 2012 and 2019, showing that the higher trypsin level used is compatible with both ACE2-dependent and ACE2-independent sarbecoviruses. Electron microscopy of virions showed that the spike density on clade 2 virions may vary from clade 1 RBD sarbecoviruses. This new sarbecovirus isolation protocol increases the chance of viral isolation from field samples and has extended our ability to explore and understand the biological features of less studied sarbecoviruses in the laboratory.

## RESULTS

### Isolation of three novel sarbecoviruses from Chinese horseshoe bats in the presence of trypsin

**Fig 1:**
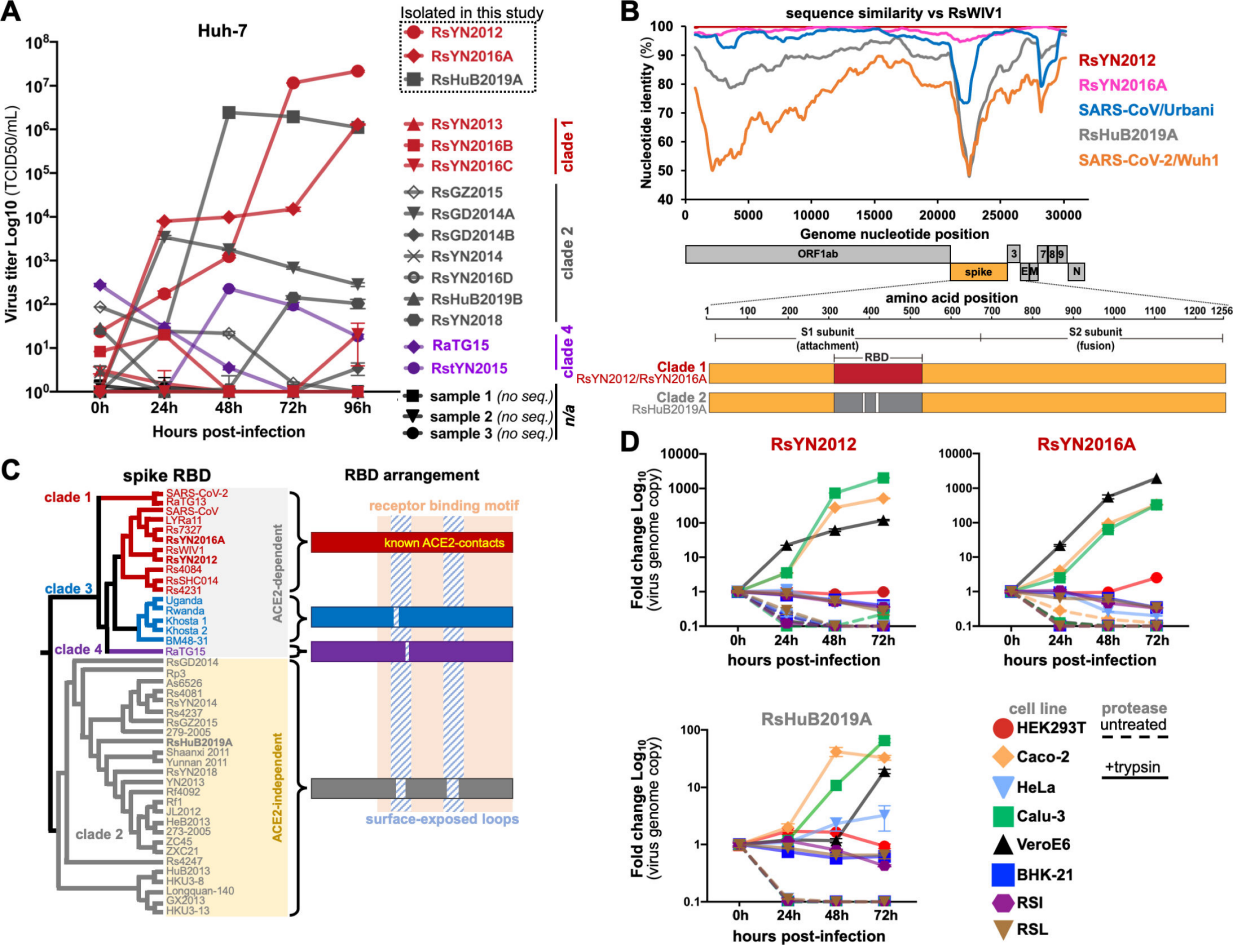
Isolation of clade 1 and clade 2 RBD sarbecoviruses on human cell lines. (**A**) Field samples were used to inoculate Huh-7 cells in the presence of trypsin. Viral titers were quantified in supernatants by qRT-PCR. (**B**) Whole-genome nucleotide sequences were compared to RsWIV1 with a sequence similarity plot. Open reading frame (ORF) positions are indicated under the *x*-axis. (**C**) Cladogram analysis of RBD amino acid sequences (corresponding to SARS-COV spike aa323–510) for sarbecoviruses. RBD indels and receptor preferences are indicated for each functional phylogenetic clade. Viruses isolated in this study are in bold font. (**D**) Viral isolates were inoculated on indicated cell cultures, and viral replication was monitored by qRT-PCR. *n/a*, not available; qRT-PCR, quantitative RT-PCR.

In our previous studies, we showed that some clade 2 sarbecoviruses are capable of entering and replicating in human cell lines in a high trypsin environment (4, 7, 13). To assess if trypsin-mediated entry is sufficient to support clade 2 virus isolation from field samples, we chose 18 bat fecal swabs or fecal samples from the Wuhan Institute of Virology (WIV) biobank, which were collected from individual bats during a 7-year longitudinal survey from 2012 to 2019. Sixteen of 18 samples tested positive for betacoronaviruses using an established reverse transcription (RT)-nested PCR targeting a fragment of the RNA-dependent RNA polymerase (RdRp) gene (Table S1) (18, 19). We also performed next-generation sequencing (NGS) on all 18 samples to obtain nearly full-length genome sequences for 14 viruses (Table S1), including two isolates, RaTG15 (sample ID: 7909) and RstYN2015 (sample ID: 7896), which we have reported previously (5). In general, samples with lower Ct values produced sequence data, while samples with higher Ct values were somewhat less consistent in our NGS pipeline (Table S1). Based on our study of recombinant bat sarbecoviruses, we modified our virus isolation protocol to include a high concentration of trypsin (100 µg/mL), cold media pre-wash step, and a chilled centrifugation step during inoculation [see Materials and Methods and reference (13)]. With this modified protocol, we isolated three sarbecoviruses from positive samples, in a human liver cell line (Huh-7) and named them: RsYN2012, RsYN2016A, and RsHuB2019A (Fig. 1A). We further examined the genome sequence of the three isolates and found that they shared a similar genome structure and organization with other bat and human sarbecoviruses (Fig. 1B). Based on the RBD portion of the spike that we and others have previously used to group sarbecoviruses into clades, RsYN2012 and RsYN2016A belong to clade 1, and RsHuB2019A belongs to clade 2 (Fig. 1A through C). Comparing whole genomes, the two clade 1 viruses were 99.9% and 98.3% similar to bat SARS-related coronavirus (SARSr-CoV), RsWIV1, while the clade 2 virus RsHuB2019A showed 93.2% nucleotide similarity with bat SARSr-CoV, HKU3-1 (Fig. 1B; Table 1). All three viruses were only approximately 80% similar to SARS-CoV-2, and less than 80% similar with clade 3 and 4 viruses (Fig. 1B; Table 1). The variable region of RsHuB2019A is in the spike genes which exhibited between 65% and 77% nucleotide similarity to the clade 1, 3, and 4 viruses (Fig. 1B; Table 1).

**TABLE 1:** 

Sequence identities with SARS-CoV, SARS-CoV-2, and related bat coronaviruses (nt/aa %)												
		Full-length genome	ORF1a	ORF1b	Spike	ORF3	Envelope	Matrix	ORF6	ORF7a	ORF7b	Nucleocapsid
RsHuB2019A	SARS-CoV	88.3	88.2/94.4	92.5/99.0	76.4/79.5	83.0/82.9	97.4/100	94.6/97.3	93.2/90.6	93.8/91.9	93.3/93.3	97.2/97.9
	SARS-CoV-2	79.6	76.0/80.9	86.1/95.5	73.1/77.9	75.3/74.2	95.2/96.1	84.2/90.1	75.3/66.1	84.2/86.9	84.1/77.3	88.7/91.0
	Bat SARSr-CoV RsWIV1	88.6	88.3/94.2	92.5/99.1	76.4/79.3	83.5/83.6	97.8/100	95.2/98.2	92.2/89.1	93.5/95.1	93.3/95.6	96.6/97.6
	Bat SARSr-CoV HKU3-1	93.2	94.0/97.3	93.2/98.7	85.5/89.2	95.3/95.3	100/100	98.5/98.6	98.4/96.9	94.9/96.7	97.0/100	96.9/97.2
	Bat SARSr-CoV BM48-31	78.6	76.8/81.6	85.6/96.1	70.2/74.4	71.6/68.6	90.5/92.2	80.5/91.0	66.1/52.4	63.3/58.0	60.2/63.4	78.6/88.0
	Bat SARSr-CoV RaTG15	74.5	71.3/76.5	83.6/94.5	65.9/68.9	70.1/66.9	85.3/81.8	79.5/92.3	67.8/55.9	63.4/54.9	51.1/33.3	78.7/88.5
RsYN2012	SARS-CoV	95.6	96.9/97.9	96.4/99.3	90.2/92.4	98.5/97.1	99.1/100	97.3/98.2	95.2/92.2	93.0/92.7	93.3/93.3	98.5/99.8
	SARS-CoV-2	79.6	76.0/80.5	85.9/95.6	73.9/78	75.9/74.2	95.6/96.1	84.8/90.1	78.0/72.6	85.5/88.5	84.1/77.3	88.5/91.0
	Bat SARSr-CoV RsWIV1	99.9	100/100	100/100	100/99.9	99.8/99.6	100/100	100/100	100/100	92.7/95.1	93.3/95.6	100/100
	Bat SARSr-CoV HKU3-1	88.2	88.1/94.2	90.9/98.6	77.7/80.1	83.0/82.5	97.8/100	94.0/96.8	93.2/89.1	96.2/96.7	97/100	96.1/96.4
	Bat SARSr-CoV BM48-31	78.9	77.0/81.4	85.7/96.2	70.9/75.8	73.4/72.7	90.9/92.2	80.3/90.5	65.1/52.4	65.3/58.8	60.2/63.4	78.5/88.0
	Bat SARSr-CoV RaTG15	74.5	71.2/76.5	83.5/94.5	65.6/70.1	70.5/66.5	85.7/100	78.9/91.4	66.7/62.7	64.8/56.6	51.1/33.3	79.0/88.0
RsYN2016A	SARS-CoV	95.6	97.0/98.2	96.5/99.4	90.2/92.5	97.6/96.4	99.6/100	97.0/98.2	94.8/92.2	94.6/94.3	95.6/93.3	98.6/99.8
	SARS-CoV-2	79.7	76.0/80.9	86.3/95.7	73.7/77.9	75.6/73.8	94.3/96.1	84.8/90.1	78.0/72.6	84.7/87.7	85.6/81.8	89.0/91.4
	Bat SARSr-CoV RsWIV1	98.3	98.8/100	98.3/99.8	96.5/98.8	98.3/98.2	98.7/100	99.7/100	99.5/100	99.7/99.2	100/100	98.7/99.5
	Bat SARSr-CoV HKU3-1	88.2	88.3/94.5	90.9/98.7	77.5/80.2	82.5/81.8	97.0/100	93.7/96.8	92.7/89.1	93.0/95.9	92.6/95.6	96.5/96.9
	Bat SARSr-CoV BM48-31	78.8	77.2/81.6	85.6/96.3	71.1/75.8	73.3/72.0	90.0/92.2	80.5/90.5	65.1/52.4	64.1/58.8	63.4/68.3	78.9/88.5
	Bat SARSr-CoV RaTG15	74.6	71.2/76.8	83.6/94.6	65.4/69.9	70.2/65.8	85.3/81.8	78.9/91.4	66.1/62.7	63.4/54.9	52.6/35.6	79.1/88.5

### Cellular tropism of the three bat sarbecoviruses

Next, we tested common laboratory cell lines that are known to support the entry and replication of several human coronaviruses. We found, in addition to Huh-7 cells, the two clade 1 viruses, RsYN2012 and RsYN2016A, could replicate efficiently in human cell lines (Caco-2 and Calu-3) and African Green Monkey cells (VeroE6) in the presence of trypsin, but poorly infected these cells in the absence of trypsin (Fig. 1D). The clade 2 virus RsHuB2019A replicated in Caco-2, Calu-3, and VeroE6 cells like clade 1 viruses in the presence of trypsin, but with lower efficiency (Fig. 1D). In addition, HeLa cells were semi-permissive for the clade 2 virus, RsHuB2019A, in the presence of trypsin (Fig. 1D). Consistent with our prior study, both clade 1 and 2 virus were unable to replicate efficiently in baby hamster kidney (BHK-21) and two bat primary cell lines, including *Rhinolophus sinicus* intestine (RSI) and lung (RSL), in the presence or absence of trypsin (Fig. 1D) (13).

### ACE2 is the receptor for RBD clade 1 but not RBD clade 2 sarbecovirus

**Fig 2:**
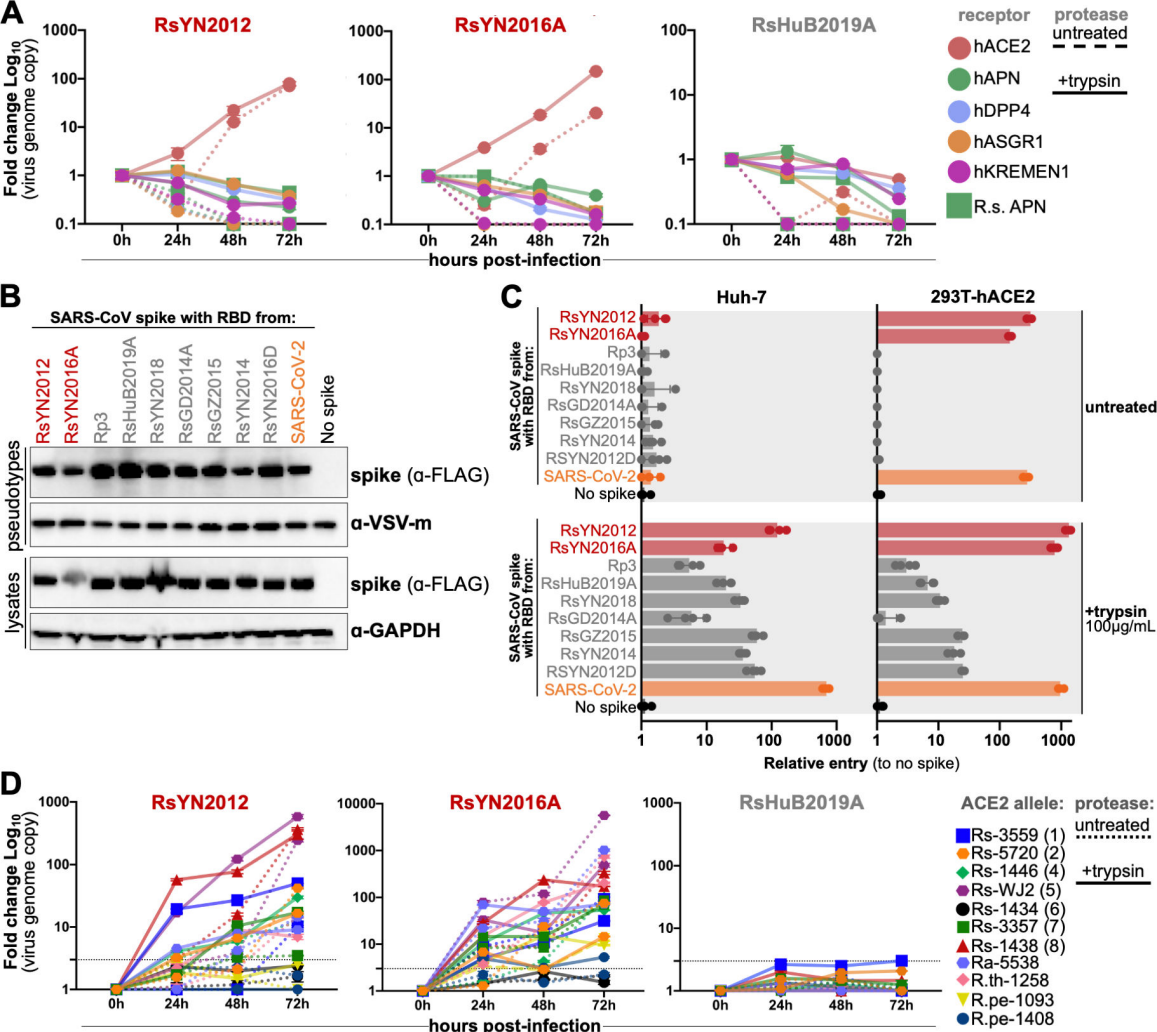
Clade 2 RBD sarbecoviruses do not use any known coronavirus receptors for cell entry. (**A**) BHK-21 cells were transfected with human orthologs of known coronavirus receptors and then infected with viral isolates. Replication was quantified by qRT-PCR. (**B**) VSV-based pseudotyped virus particles bearing chimeric SARS-CoV spikes with the indicated virus RBDs were generated in HEK 293T cells and concentrated in OptiPrep. Spike was detected in cell lysates and pseudotyped virus particles by probing for FLAG. (**C**) Huh-7 cells or cells transduced to express human ACE2 were infected with pseudotyped virus particles, and luciferase was measured as a readout for cell entry. (**D**) BHK-21 cells were transfected with the indicated bat ACE2 alleles and infected with viral isolates. Replication was monitored by qRT-PCR.

To explore the receptor usage of the three novel bat sarbecoviruses, we performed virus infectivity studies using BHK-21 cells expressing known coronavirus receptors from humans and bats. Consistent with prior studies (4, 6, 13), we found that only the clade 1 virus could utilize human ACE2 for cell entry and that the clade 2 virus, RsHuB2019A could not use any known coronavirus receptor, with or without trypsin (Fig. 2A).

To assess the cell entry capacity of the viruses we failed to isolate from the other samples, we assembled a panel of recombinant RBD chimeras, with SARS-CoV chimeric spike containing the RBD sequence from the different samples (4). Of the eight clade 2 virus-positive samples we attempted to isolate virus from, four samples contained newly identified viruses (samples B228, 190366, 141341, and 151491; Table S1), while the RBD sequences in the other samples were identical to other RBDs from this study or RBD sequences we have previously tested (Rs4081 and As6526; Table S1) (4, 5, 7, 13). The RBDs for clade 1 viruses RsYN2012 and RsYN2016A are identical to RBDs from RsWIV1 and Rs7327, respectively, which we have also previously tested (4). For comparison, we included a SARS-CoV spike chimera with the RBD from SARS-CoV-2 and a clade 2 RBD from the prototypical virus, Rp3 (Fig. 2B and C) (10). All RBD chimeras exhibited similar levels of incorporation into VSV-based pseudotyped virus particles (Fig. 2B). We have previously shown exogenous trypsin allows mediated sarbecovirus entry into otherwise poorly susceptible cell lines, Huh-7 and HEK 293T (4, 7, 13). Transduction of HEK 293T cells with human ACE2 allows for clear detection of ACE2-dependent entry (10, 11). Consistent with the live virus infection assay results, only pseudotyped virus particles with clade 1 virus RBDs were capable of entering and transducing human ACE2 expressing cells without trypsin, but not any of the clade 2 viruses (Fig. 2C). As we have shown for other clade 2 RBDs, the addition of trypsin dramatically increased entry for these spikes, with a notable exception for the RBD from RsGD2014A (Fig. 2C).

Previous studies from our groups and others have reported that the ACE2 gene is diverse across bat species (12, 20 – 23). We have shown that the ACE2 gene is highly polymorphic in Chinese horseshoe bats (*R. sinicus*), and that sarbecovirus entry is specific for only some of these alleles (20). To further confirm if the ACE2 orthologs from different bat species or different Chinese horseshoe bat (*R. sinicus*) ACE2 alleles support the entry of clade 2 viruses, we tested a large panel of bat ACE2 alleles for their ability to support live virus infection in BHK-21 cells. Consistent with our previous study (20), we found the clade 1 virus, RsYN2012 and RsYN2016A, could utilize most alleles from *R. sinicus* ACE2, as well as ACE2 from *Rhinolophus affinis* and *Rhinolophus thomasi*, for cell entry regardless of trypsin (Fig. 2D). RsYN2016A could also enter the BHK-21 cell expressing *Rhinolophus pearsonii* (R.pe) ACE2-1093 with low efficiency, but not the allele 1408. However, RsYN2012 could not use either of the ACE2 alleles from *Rhinolophus pearsonii* for entry (Fig. 2D). In contrast, we found none of these bat ACE2 genes supported replication of clade 2 virus RsHuB2019A, even in the presence of trypsin (Fig. 2D). Taken together, these results demonstrate that only clade 1 viruses we isolated possess the capacity to use the ACE2 from different species and that the clade 2 virus employs an unknown molecule(s) for entry in human cells that is distinct from other coronaviruses.

### Tissue culture adaptations reduce ACE2-independent spike degradation by trypsin

**Fig 3 F3:**
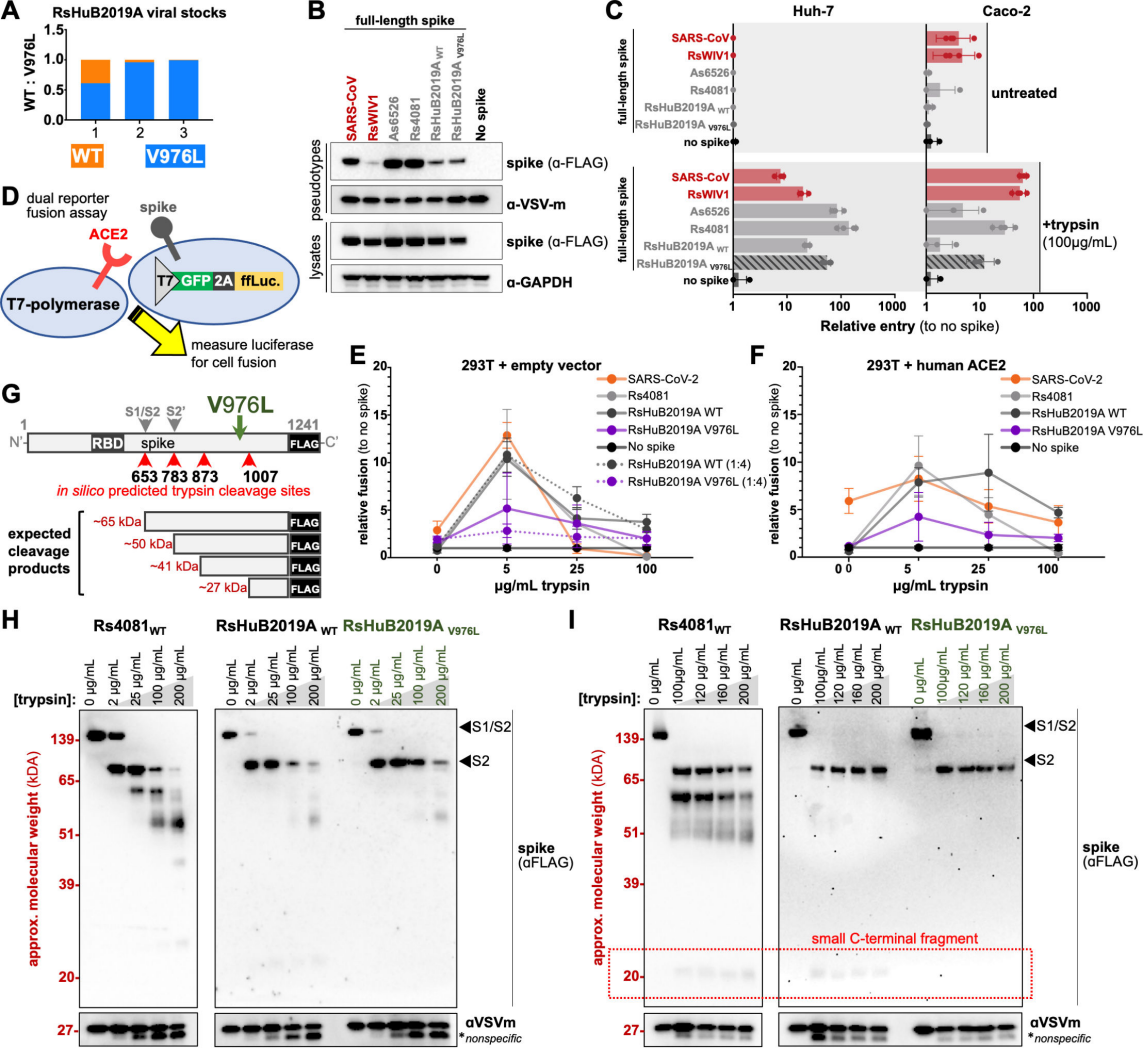
Clade 2 RBD virus adaptation to the cell culture. (**A**) V976L mutation emerged in RsHuB2019A virus stocks. (**B**) Pseudotyped virus particles were produced with full-length spike wild type (WT) or the V967L mutant. Spike was detected in producer cells and pseudotyped virus particles by western blot for FLAG. (**C**) Indicated cells were infected with pseudotyped virus particles in the presence or absence of trypsin. (**D**) Schematic overview of the dual-reporter fusion assay developed for this study. T7 polymerase drives the expression of GFP and luciferase separated by self-cleaving 2A peptide from porcine teschovirus-1 (P2A). (**E**) HEK 293T cells expressing receptor or (**F**) empty vector and T7 polymerase were combined with cells expressing spike and the T7-driven reporter. Luciferase was measured as a readout for cell fusion. Dotted lines indicate data from 1:4 ratio of receptor: spike cells. (**G**) Overview of RsHuB2019A spike with *in silico* predicted trypsin digest sites indicated. Location of V976L is indicated in green. (**H**) Concentrated pseudotyped virus particles were combined with a wide range of trypsin dilutions or (**I**) a fine range of trypsin dilutions and incubated at 37°C. Spike digestion was assessed by western blot for the FLAG epitope.

**Fig 4:**
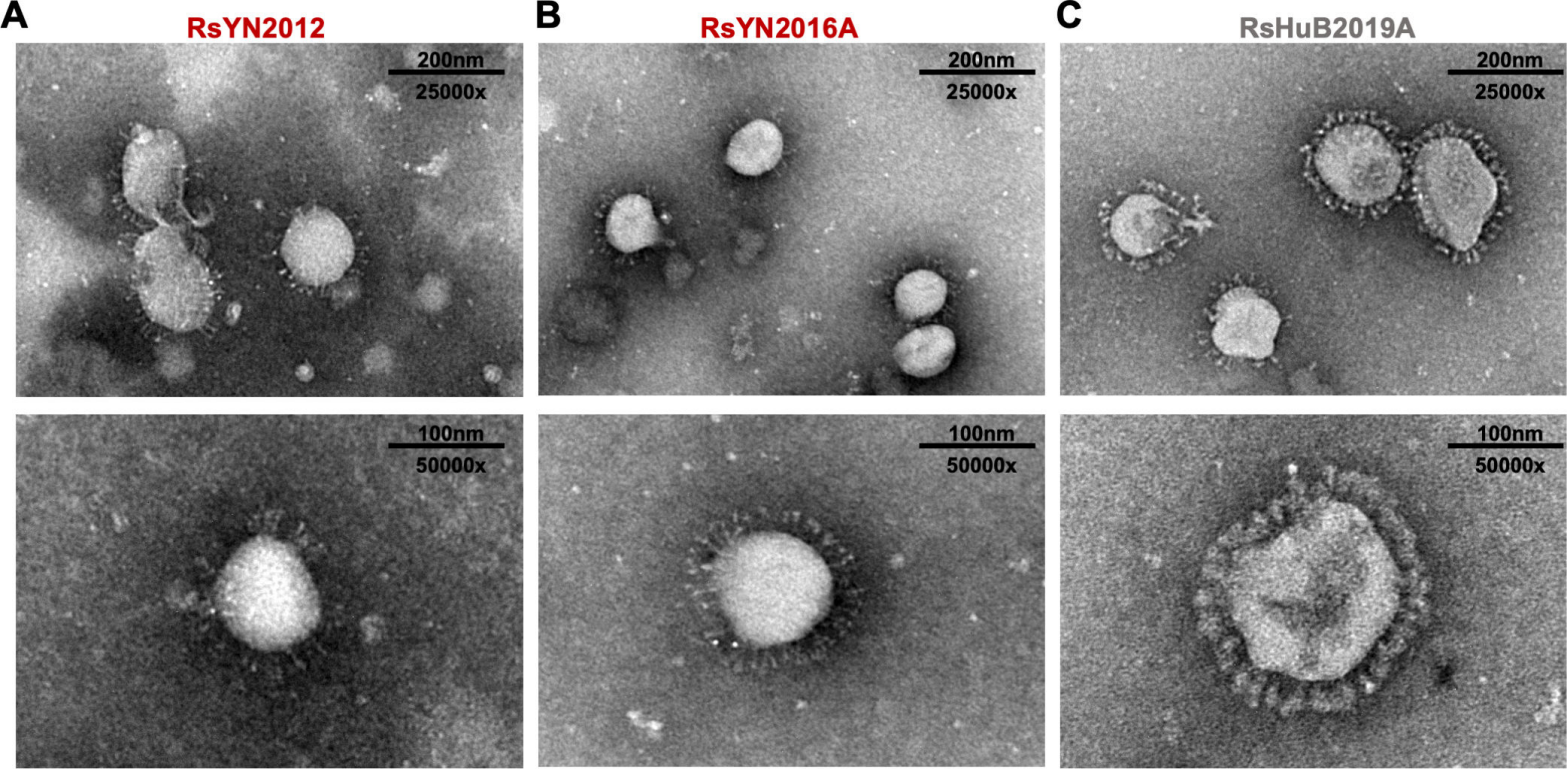
Electron microscopy of purified viral isolates. Viral stocks for (**A**) RsYN2012, (**B**) RsYN2016A, or (**C**) RsHuB2019A were visualized by transmission electron microscopy. The bottom images were taken at a higher magnification to show detail.

Coronavirus spike genes are known to rapidly acquire cell-culture-specific adaptations sometimes in as few as three passages (24 – 31). Over the course of this study, we replenished our viral stocks by subsequently passaging the previous stock in Huh-7 cells, leading to three viral passages (experiments from Fig. 1 are passage 1, Fig. 2 are passage 2, and Fig. 4 are passage 3). We extracted viral RNA from the remainder of each stock after each passage and looked for potential cell-culture adaptations, across the whole viral genome by NGS. We found one nonsynonymous (T24550G) substitution that emerged at low frequency in the clade 2 virus, RsHuB2019A at the first passage, resulting in V976L mutation in the S gene. By the third passage, we observed an increase in the frequency of spike V976L mutation (from 61% to 99.4%) with L976 becoming the dominant polymorphism (Fig. 3A). We did not observe additional mutations elsewhere in the RsHuB2019A genome or in the genomes of the clade 1 RBD viruses, RsYN2012 and RsYN2016A.

To characterize the V976L mutation in the RsHuB2019A spike, we constructed VSV-based pseudotyped virus particles containing full-length spikes with either V976 or L976 and tested their cell entry in Huh-7 and Caco-2 cell lines. Full-length spikes from clade 1 viruses, SARS-CoV, and RsWIV1, as well as clade 2 viruses, Rs4081 and As6526, were used as comparative controls (13) (Fig. 3B and C). We found that V976L mutation did not increase spike incorporation into virions (Fig. 3B; Fig. S1A) but moderately enhanced the entry of RsHuB2019A in both Huh-7 and Caco-2 cell lines, only in the presence of trypsin (Fig. 3C; Fig. S1B). Introducing a similar mutation in RsWIV1 spike (V991L), which is 99% identical to the RsYN2012 spike, also resulted in higher pseudotyped virus particle entry without affecting expression or incorporation (Fig. S1A and B).

Because the V976L mutation is in close proximity to the host cell fusion machinery present in the spike S2 domain, we wondered if this mutation was influencing the fusogenic properties of RsHuB2019A spike. To test if this mutation modulated spike cell fusion properties, we performed a cell-cell fusion assay similar to previous approaches by combing cells individually expressing spike or receptor and a complementary reporter system (32). HEK 293T cells expressing T7 polymerase and human ACE2 or empty vector were combined 1:1, with HEK 293T cells expressing a T7-driven reporter cassette and spike (Fig. 3D). Because RsHuB2019A spike had reduced incorporation into pseudotyped virus particles (Fig. 3B), we also included a condition with four times the amount of spike-containing cells to receptor cells (Fig. 3E, **1:4, dotted line**). Increasing the concentration of trypsin to even 5 µg/mL resulted in more than a 10-fold increase in cell fusion for spikes with clade 1 and clade 2 RBDs, while the addition of human ACE2 to the cells increased basal entry of SARS-CoV-2 spike without trypsin (Fig. 3E and F). Notably, regardless of the ratio between spike-expressing cells and target cells, viral fusion was reduced for the RsHuB2019A spike with V967L mutation compared to the wild-type spike (Fig. 3E and F, **dotted lines**). In contrast, ACE2-dependent RsWIV1 spike with the V991L mutation demonstrated enhanced fusion in a similar assay (Fig. S1C and D).

To further explore how the V976L mutation in increased spike cell entry in the presence of trypsin, we tested the *in vitro* trypsin resistance of spike, with the clade 2 virus Rs4081 as control. Purified V976 or L976 pseudotyped virus particles were combined with different amounts of trypsin, incubated at 37°C for 5 minutes, and spike degradation was analyzed by western blot. As we have previously shown, trypsin cleaved the Rs4081 spike into several fragments, including the expected fragments corresponding to cleavage at the S1/S2 boundary as well as a secondary, S2′ site, at 25 µg/mL or above trypsin (13) (Fig. 3G and H). In contrast, the RsHuB2019A spike displayed less of these degradation products, with the V976L mutation showing resistance to trypsin digestion at 100 µg/mL—the concentration we used to propagate the virus in our cultures (Fig. 3H). When we performed the second trypsin digestion between 100 and 200 μg/mL and used a more sensitive western blot substrate, a smaller digestion product, the approximate size of a C-terminal fragment of spike that is predicted to digest from a site near V976, was absent from the V976L mutant but present for Rs4081 and wild-type RsHuB2019A spike (Fig. 3G and I, **boxed in red**). Thus, V967L may reduce trypsin digestion in spike near this mutation. Taken together, these findings strongly suggest the clade 2 virus spike adapted to the exogenous (porcine) trypsin included during viral propagation, rather than the cell lines themselves.

### Electron microscopy of purified virions reveals potential differences between RBD clades

To confirm if we had isolated the three sarbecorviruses successfully, we purified viral stocks over a 30% sucrose cushion and processed the samples for analysis by transmission electron microscopy. Purified viral particles displayed typical coronavirus morphology under electron microscopy: virions are approximately 100–120 nm in diameter, with “corona-like” ring of spike glycoproteins at the surface. Interestingly, the glycoprotein layer on clade 2 virions appeared denser than on clade 1 RBD virions (Fig. 4A through C; Fig. S2A through C).

## DISCUSSION

Although hundreds of sarbecoviruses have been discovered in animals, more than two-thirds of these viruses have clade 2 RBD spikes, which contain indel mutations that prevent them from using host ACE2 as a cell receptor (2 – 4, 6, 11, 12). Attempts to isolate these ACE2-independent sarbecoviruses from field samples have failed, hampering downstream laboratory-based assessments and leading to the general assumption that they pose little threat to humans. However, we have demonstrated the RBDs from a small group of these viruses are capable of mediating human cell entry, which we have verified with whole spike proteins and most recently, complete sarbecovirus replication recovered through reverse genetics (4, 7, 13). Here, we developed a virus isolation protocol built on these findings that is suitable for recovering both ACE2-dependent and -independent sarbecoviruses from bat fecal samples, underscoring the broader zoonotic threat posed by sarbecoviruses and the complexities underlying coronavirus cell entry.

Successful isolation of both ACE2-dependent and -independent viruses using the same protocol suggests this approach is broadly applicable for sarbecoviruses, and an improvement over existing sarbecovirus isolation protocols, which have only isolated ACE2-dependent viruses. Notably, we isolated a viable virus (RsYN2012) from a field sample that had been in storage for more than 10 years (Table S1). The viruses we isolated were from samples with some of the lowest Ct values of the samples tested, suggesting higher viral titers are ideally required for successful isolation (Table S1). Although we failed to recover the sample RsGD2014A, which had a low Ct value, the RBD from this virus was unable to mediate efficient viral entry in our pseudotype assays suggesting this virus is not as compatible with human cells as RsHuB2019A (Table S1; Fig. 2C).

The ACE2-dependent viruses we isolated, RsYN2012 and RsYN2016A, were strikingly similar to two other sarbecoviruses we have previously isolated or tested: RsWIV1 and Rs7327 (Fig. 1B; Table 1) (4, 19). RsWIV1 and RsYN2012 were collected from the same location and time during the same sampling mission, which likely explains this close similarity (Table S1). However, the high similarity observed between RsWIV1 and viruses collected at later time points, including RsYN2016A, suggests evolutionary constraints on these viruses in their hosts.

Coronaviruses acquire mutations when grown in cell culture and can rapidly adapt to the conditions and cells used for their propagation (24 – 31). Sequencing the viral stocks produced for this study revealed the emergence of a tissue-culture adaptation in the clade 2 virus, RsHuB2019A, which appeared to increase cell entry in pseudotyped virus particle entry experiments (Fig. 3A through C; Fig. S1B). Since the spike-V976L mutation is close to known fusion machinery in the spike-S2 region, we tested spike fusion similar to other studies (32 – 34). Curiously, we found that this spike mutation resulted in lower fusion efficiency compared to the wild-type RsHuB2019A spike protein (Fig. 3D through F), while introducing a similar mutation in the ACE2-dependent spike, RsWIV1, resulted in higher cell fusion by the same assay (Fig. S1C and D). This contrast in fusion activity between RsWIV1 and RsHuB2019A spikes may be due to the inherent differences between the ACE2-spike interface and the interface between ACE2-independent spikes with “receptor X.” Alternatively, this discrepancy may be from the different expression levels of ACE2 and “receptor X” used for the assay. While we can provide 293T cells with human ACE2 in *trans*, thereby ensuring high levels of both spike and receptor, we currently do not know the receptor for ACE2-independent spikes like RsHuB2019A and are thus unable to modulate the receptor levels on the target cells. A spike protein that prematurely assumes fusion form in a receptor-rich environment may perform worse than a spike protein that is less fusogenic and has more appropriate-timed processing. However, if the receptor levels are low or the affinity between spike and receptor is weak, then a more fusogenic spike may have the advantage over the less fusogenic spike. Our entry assay results suggest that 293T cells have lower levels of the unknown receptor for RsHuB2019A than Huh-7 (Fig. 1D and Fig. 2C). Efficient spike-mediated cell fusion depends on coordinated spike processing after receptor engagement; therefore, we may be unable to detect the true effects of fusion without increasing the receptor levels (35). Ideally, these experiments should be repeated when the receptor for clade 2 viruses has been identified and can be provided in high levels, in *trans*.

To understand the effects of this mutation more directly, we assessed the trypsin degradation of spike proteins (Fig. 3A through I [Fig F3]). A close inspection of western blots following trypsin treatment of concentrated pseudotyped virus particles revealed this mutation resulted in the loss of a low-molecular-weight digestion product, suggesting the mutation enhanced spike resistance to the trypsin used in our protocol (Fig. 3). Taken together with our entry and fusion results, the RsHuB2019A-V976L mutation appears to prevent harmful digestion of the spike near the fusion machinery, which likely prevents the spike from assuming a fusion intermediate form before engaging the receptor (Fig. 3; Fig. S1). The trypsin we used in our studies is porcine-derived and not treated with L-1-tosylamido-2-phenylethyl chloromethyl ketone (TPCK), which may allow for additional spike digestion compared to TPCK-treated trypsin. Importantly, RsHuB2019A spike V967L still required trypsin for entry into cells (Fig. 3C), suggesting that the clade 2 viruses may not be capable of readily “evolving away from” trypsin dependence. Thus, while our protocol is suitable for the isolation of sarbecoviruses, more studies are needed into the species-specific proteases utilized by these viruses, which may lead to further protocol changes that reduce the development of cell-culture adaptations.

Electron microscopy of clade 1 and clade 2 virus isolates revealed a potential difference in the spike corona surrounding each virion. The spike trimers on ACE2-dependent clade 1 viruses appeared thinner and less evenly distributed than clade 2 virions, which may help explain clade 1’s increased sensitivity to trypsin versus clade 2 viruses (Fig. 4; Fig. S1). The virus stocks used for electron microscopy contained trypsin at the time of processing; therefore, the differences in the fullness of the spike corona may reflect the overall trypsin resistance we have previously noted for the clade 2 RBD spikes (13). As the virus stocks used in our electron microscopy are from a later passage, we cannot exclude the possibility that this distinction may also derive from the presence of spike mutation V976L in RsHuB2019A.

Other betacoronaviruses may provide clues about the entry mechanisms for clade 2 sarbecoviruses. For example, the bat merbecoviruses, PDF2180 and NeoCoV, contain RBD deletions that prevent them from using host dipeptidyl peptidase IV (DPP4 as their receptor and have been shown to require trypsin for their cell entry and propagation in human cell cultures (15). However, a recent study has shown these viruses bind to host ACE2 as a receptor and that providing this receptor can effectively remove the protease requirement (36). While we and others have shown the clade 2 sarbecoviruses do not use any known coronavirus receptor, our studies strongly suggest these viruses do rely on a conserved host molecule for entry (Fig. 2) (4, 6, 7, 11 – 13). Thus, more studies are needed to identify the receptor for clade 2 sarbecoviruses. Taken together, our viral isolates demonstrate a cluster of bat sarbecoviruses that can infect human cells using mechanisms distinct from known human sarbecoviruses.

## MATERIALS AND METHODS

### Cells

HEK 293T, HEK 293T/17, BHK-21, VeroE6, Calu-3, and HeLa were obtained from the American Type Culture Collection (ATCC), and Caco-2 was generously gifted by Prof. Qin-Xue Hu. Bat-derived cell lines RSI and RSL were stored at the WIV as described previously (13, 37). HEK 293T, HEK 293T/17, BHK-21, VeroE6, Huh-7, and HeLa were maintained in Dulbecco’s modified Eagle medium (DMEM) supplemented with 10% fetal bovine serum (FBS). Calu-3, RSI, and RSL were maintained in DMEM/Nutrient Mixture F-12 supplemented with 15% FBS. Cultures were maintained at 37°C with 5% CO_2_. All cell lines used in this study were species verified by cytochrome sequencing and tested negative for mycoplasma contamination by PCR as described previously (4, 38).

### Plasmids

Expression plasmids for human ACE2, human DPP4, human APN, human ASGR1, human KERMEN1, *Rhinolophus sinicus* APN, *Rhinolophus affinis* ACE2, and different alleles of *Rhinolophus sinicus* ACE2, were described previously (13, 20). *Rhinolophus pearsonii* ACE2-1093 and 1408, and *Rhinolophus thomasi* ACE2 were amplified from the bat intestine as described previously (20). The spike or RBD coding sequences for SARS-CoV/-2, RsWIV1, Rs4081, As6526, Rp3, RsYN2012, RsYN2016A, RsYN2018, RsGD2014A, RsGZ2015, RsHuB2019A, and RsHuB2019A-S-V976L were codon optimized for human cells as previously described (13). Plasmid encoding for a green fluorescent protein (GFP) and luciferase dual-reporter cassette under a T7-promoter was generated by cloning the firefly luciferase gene downstream of GFP in pUC19-T7-IRES-GFP. pUC19-T7 pro-IRES-EGFP was a gift from Fei Chen (Addgene plasmid no. 138586; http://n2t.net/addgene:138586; RRID: Addgene_138586). All the plasmids used in this study were verified by Sanger sequencing or next-generation whole plasmid sequencing (Plasmidsaurus Inc.).

### Virus isolation

Bat fecal swabs or fecal samples were collected from several provinces in China over a 7-year period and stored at −80°C as described previously (19). The bat species was confirmed by cytochrome b sequence analysis as described previously (19). For virus isolation, the fecal samples were thawed on ice and centrifuged at 10,000×*g* for 10 minutes at 4°C before use. The supernatant (in 200 µL buffer) was filtered through 0.45-µm membranes and diluted 1:2 with cold DMEM, and trypsin was added to a final concentration of 625 µg/mL. Trypsin used for virus propagation was standard cell culture grade 0.25% porcine trypsin without EDTA and phenol red (Thermo Fisher Scientific). Huh-7 cells in 24-well plate format were washed once with DMEM before incubating with 300 µL of sample and trypsin. Inoculated plates were centrifuged at 1,200×*g* at 4°C for 1hour, then incubated at 37°C overnight. Approximately 20–24 hours post-infection, the monolayer cells were supplied with 300 µL of fresh DMEM with 4% FBS and incubated at 37°C for 96hours. Cell-free supernatant was collected daily for virus detection by RT-PCR.

### Pseudotyped virus particle production and entry assay

The Vesicular Stomatitis Virus (VSV)-based coronavirus spike pseudotyped virus particle entry assays were performed as previously described with minor adjustments (4, 7, 10, 13). In brief, target cells were seeded in a 96-well plate and washed with phosphate-buffered saline (PBS) once before inoculating with equivalent volumes of pseudotyped virus particle stocks in the presence or absence of trypsin. Inoculated plates were centrifuged as described above. Entry efficiency was quantified 18–20 hours post-transduction by measuring the luciferase activity using Bright-Glo luciferase reagent (Promega), following manufacturer’s instructions. Relative entry was calculated as the fold-entry in relative luciferase unit over the no spike control. All experiments were performed at least three times in triplicate.

### Cell-cell fusion assay

HEK 293T cells were seeded in a six-well format. One group of cells was transfected with equivalent amounts of human ACE2 plasmid or empty plasmid and T7-polymerase plasmid. The second group of cells was transfected with equivalent amounts of spike expression plasmid and the dual reporter construct. Twenty-four hours post-transfection, cells were trypsinized, diluted to 1 × 10^6^ cells/mL, and combined in either 1:1 or 1:4 ratios (receptor:spike transfected cells). Twenty-four hours post-combining, cells were washed in cold PBS, and the cell culture media was replaced with trypsin media and subsequently centrifuged at 1,200×*g* at 4°C for 1hour (to mimic the spin-infection procedures used in the infection assays). Twenty-four hours post-trypsin treatment and centrifugation, luciferase was measured on a plate reader using the Bright-Glo luciferase reagent (Promega).

### Electron microscope imaging

Virion concentration, purification, and negative staining were performed as previously described with minor adjustments (19). In brief, fresh virus stocks were harvested at 72hours post-infection, then centrifuged at 5,000×*g* for 30 minutes at 4°C. Cell-free supernatants were collected and fixed by 0.1% formaldehyde at 4°C overnight. Inactivated virions in the supernatant were loaded onto 5 mL of 30% sucrose in PBS buffer and centrifuged at 25,000 rpm in the SW28 rotor at 4°C for 2.5hours. The pelleted virions were suspended in cold PBS, then applied to the grids and stained with 2% phosphotungstic acid (pH = 7.0) on ice. The specimens were examined using a Tecnai transmission electron microscope (FEI) at 200 kV. Images were taken at a magnification of 25,000× and 50,000×.

### Phylogenetic analysis

Routine sequence management and analysis were carried out using DNAStar. Sequence alignments were created using Clustal W in MegAlign (DNAStar). Maximum likelihood trees with sarbecovirus spike RBD amino acid sequences were generated using PhyML 3.0 (39) with 1,000 bootstrap replicates (40) and visualized as a cladogram in FigTree v1.4.4 (https://github.com/rambaut/figtree), as previously described (4, 10). Sequence similarity plot was generated using whole genomes for RsWIV1, SARS-CoV/Urbani, SARS-CoV-2, and isolates from this study using Simplot with the Kimura model, a window size of 1,500 base pairs and a step size of 150 base pairs (GenBank accession numbers: KF367457.1, AY278741.1, NC_045512.2).

### Viral replication detected by real-time RT-PCR

For replication experiments, target cells were seeded in 24-well plates and washed with DMEM before inoculating with virus stocks in the presence or absence of trypsin. For receptor usage assays, BHK-21 cells were transfected with plasmids expressing different receptors 18–20 hours before infecting with the authentic virus, in the presence or absence of exogenous trypsin. The inoculated plates were centrifuged at 1,200×*g* at 4°C for 1hour and further incubated in a 37°C incubator for 72hours. Cell-free supernatants (50 µL each time) were collected at 0, 24, 48, and 72hours post-infection and stored at −80°C for future use. Viral RNA was extracted and subjected to RT-PCR as previously described (13). Viral replication was quantified by RT-PCR using primers targeting the RdRp gene, forward primer: 5′-TTGTTCTTGCTCGCAAACATA-3′; reverse primer: 5′- CACACATGACCATCTCACTTAA-3′. The RNA from RsWIV1 stocks with known titers was used as a standard control to correlate the cycle threshold (Ct) value and virus titer of the other viruses. All samples were analyzed in duplicate on two independent runs. One representative data set is shown.

### Western blot

To check for cell expression of the spike, HEK 293T cells producing pseudotyped virus particles were lysed in 1% SDS lysis buffer, clarified by centrifugation, and blotted for FLAG as described previously (4). To check for spike incorporation, pseudotyped virus particle stocks were concentrated over a 10% OptiPrep cushion in PBS at 21,000×*g* at 4°C for 2hours and blotted for FLAG on a 10% Bis-Tris gel (Thermo Fisher Scientific) (4). Spike degradation was measured as in reference (13), whereby concentrated pseudotyped virus particle stocks were incubated with trypsin concentrations at 37°C for 5 minutes, boiled, and blotted for FLAG (13). The substrate used in Fig. 3H is SuperSignal Western Blot Substrate Pico (Thermo Fisher Scientific), and for increased sensitivity in Fig. 3I, SuperSignal Western Blot Substrate Atto (Thermo Fisher Scientific) was used as the substrate.

### Statistical analysis and graphing

All graphed data are three technical replicates that are representative of at least three biological replicates. Graphed data were analyzed and visualized in GraphPad Prism version 9.

### Biosafety and biosecurity

Laboratory work with VSV-based pseudotyped virus particles in mammalian cell lines was performed according to standard operating procedures (SOPs) under biosafety level 2 conditions that were approved by institutional biosafety committees (IBCs) at Washington State University and WIV. Work with bat SARS-related CoV was approved by the WIV IBC for this SOP and performed in WIV facilities. WIV facilities for this work adhere to the safety requirements recommended by the China National Accreditation Service for Conformity Assessment.

## Data Availability

The nearly full-length genome sequences of SARSr-CoVs obtained in this study have been deposited in the GenBank database and the accession numbers are OQ503495-OQ503506, respectively. The accession numbers of partial RdRp and spike sequences of RsYN2016D (sample ID: 160665) obtained in this study are OR364995 and OR364996. The accession number of Rhinolophus pearsonii ACE2-1093 and 1408, Rhinolophus thomasi are OQ511289-OQ511291.

## SUPPLEMENTAL MATERIAL

Supplemental information jvi.00395-23-s0001.docx Table S1 and Fig. S1 and S2.

ASM does not own the copyrights to Supplemental Material that may be linked to, or accessed through, an article. The authors have granted ASM a non-exclusive, world-wide license to publish the Supplemental Material files. Please contact the corresponding author directly for reuse.
