# Supplementary material for: Isolation of ACE2-dependent and -independent sarbecoviruses from Chinese horseshoe bats
Source: J Virol. 2023 Sep 28;97(9):e00395-23. doi: 10.1128/jvi.00395-23 (PMC10537568; doi:10.1128/jvi.00395-23)
Supplement: Supplemental information — Table S1 and Fig. S1 and S2. [file jvi.00395-23-s0001.docx]

**Guo et al. 2023**

**SUPPLEMENTARY INFORMATION**

**
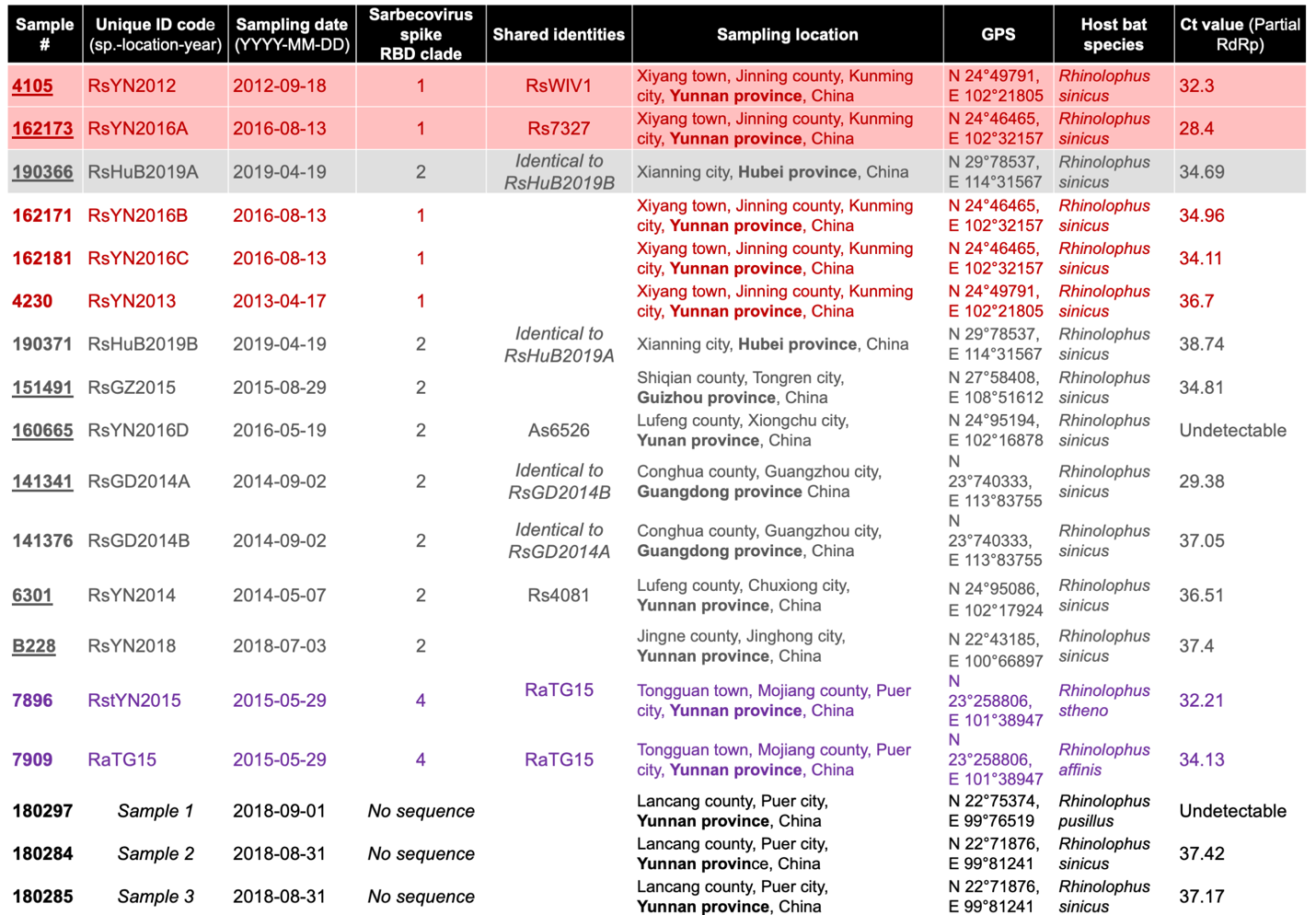
Supplementary table 1.** Metagenomic information regarding the samples from this study with isolated viruses in highlighted rows and viruses for pseudotyped virus particle experiments underlined.

**
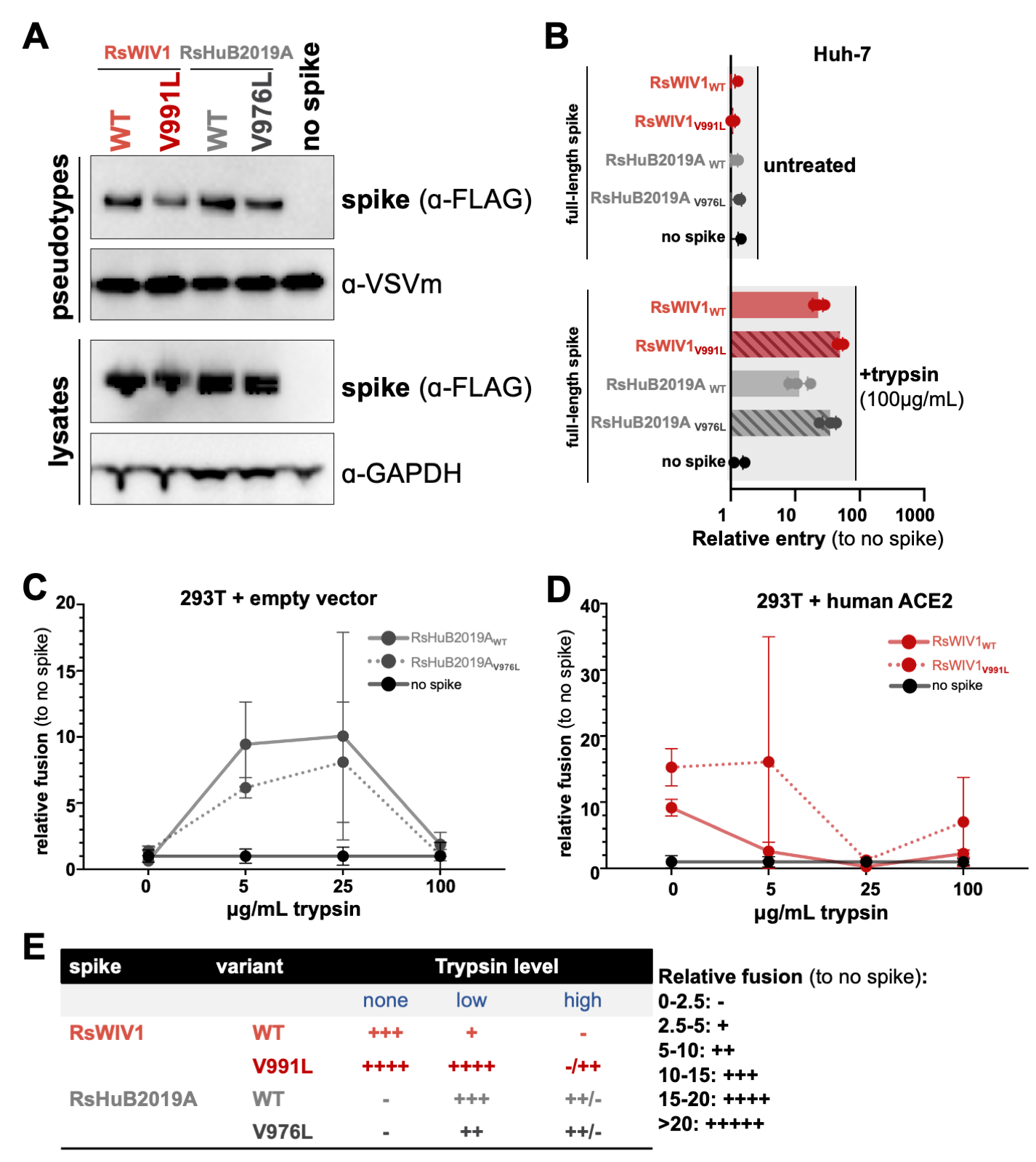
**

**Supplementary figure 1. Testing V976L equivalent mutation in ACE2-depdenent spike.** (**A**) Spike and corresponding mutant expression in HEK 293T producer cells and incorporation into concentrated pseudotyped virus particles (**B**) Huh-7 cells were infected with pseudotyped virus particles with or without trypsin. (**C**) HEK 293T cells overexpressing spike protein were combined with HEK 293T cells transfected with empty vector or (**D**) human ACE2 plasmid and incubated with trypsin. Resulting luciferase was measured for cell fusion. (**E**) Summarized results from C-D.

**
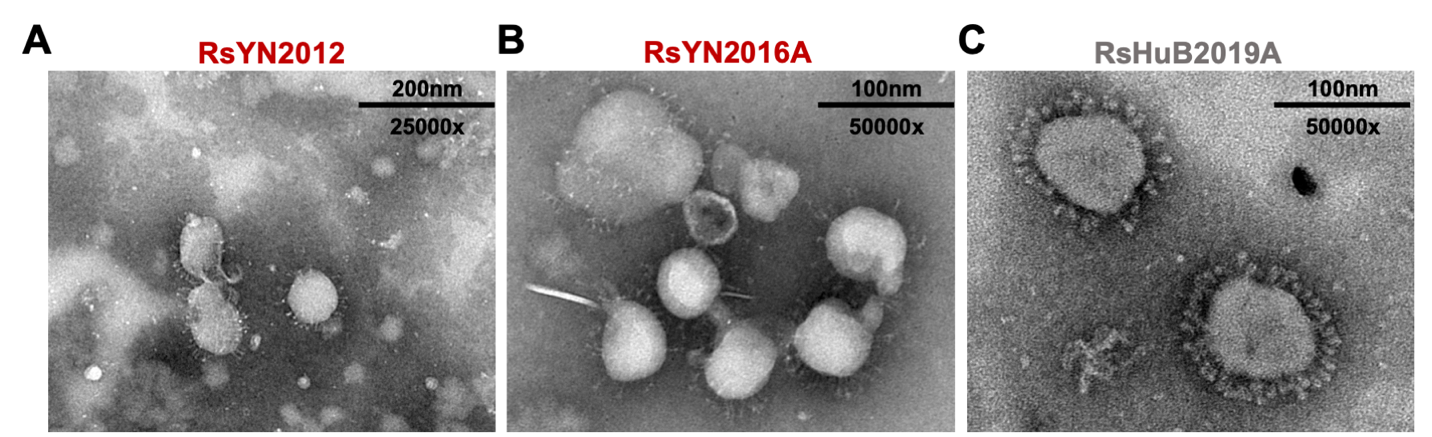
**

**Supplementary figure 2. Additional electron micrographs of purified viral isolates.** (**A**) RsYN2012 (**B**) RsYN2016A or (**C**) RsHuB2019A were visualized by transmission electron microscopy.
